# Menstrual cycle disorders as an early symptom of energy deficiency among female physique athletes assessed using the Low Energy Availability in Females Questionnaire (LEAF-Q)

**DOI:** 10.1371/journal.pone.0303703

**Published:** 2024-06-07

**Authors:** Joanna Witkoś, Edyta Luberda, Grzegorz Błażejewski, Ewa Strój

**Affiliations:** Faculty of Medicine and Health Science, Andrzej Frycz Modrzewski Krakow University, Kraków, Poland; Università degli Studi di Milano: Universita degli Studi di Milano, ITALY

## Abstract

Physique competitions are weight-sensitive sports in which stage presentation, aesthetic appearance and posing ability of the athletes are judged rather than physical performance. The aim of this study was to assess low energy availability among female physique athletes by using the LEAF-Q. The study involved 104 females who were physique athletes. Monthly cycle disorders were reported in 58.65% of the women, that is periods stopped for three consecutive months or longer (amenorrhea). This situation occurred before the research was conducted in 43.27% of athletes and during the research in 15.38%. The physique athletes claimed that menstruation changes occurred when there was an increased exercise intensity, frequency or duration. Absence from training due to injury was reported by 27.88% of the women. The LEAF-Q identified 46.15% of the physique athletes as at risk (score ≥ 8) of low energy availability and the physiological consequences related to RED-S. Women who had menstrual cycle disorders were younger and did more training per week. Among women with menstrual disorders, cramps or stomach ache which cannot be related to menstruation occurred more frequently (p = 0.004). Absence from training or lack of participation in competition due to injuries occurred more frequently in the score ≥ 8 group (p = 0.024) thank the ≤ score 8 group. In the score ≥ 8 group menstruation changes, that is less bleeding or cessation of menstruation (p = 0.035), occurred more frequently when there was an increase in exercise intensity, frequency or duration (p = 0.002).

## Introduction

The popularity of women’s female physique athletes (Female Physique—FP) is increasing every year. FP gained recognition as a sport at the International Federation of Body Building and Fitness in Cairo in 2002 [1]. Every year, the federation hosts athletes in 196 affiliated countries. In 2017, about 1,300 male and female athletes competed at the World Fitness Championships [2]. These sports go under many names and categories such as women’s fitness, bikini fitness, wellness fitness, figure fitness and women’s athletic fitness. In these sports, body shape and proportions are judged first and foremost, but the judges also look at the health and beauty of the skin and the ability to perform on stage [1]. Despite their growing popularity, FPs have been criticised for promoting unrealistic standards for the ideal female body shape which are achieved by the female athletes, through, among others things, radical dietary manipulation [2].

FP athletes strive to achieve peak form for one or more days of competition, and often for the entire competition season, showcasing a lean and muscular body composition in multiple competitions at short intervals [3]. In order to compete successfully, women preparing for competitions place particular emphasis on maximum fat reduction with minimal loss of muscle mass. They use prolonged periods of energy restriction (a negative calorie balance) and intensive exercise programmes to achieve and maintain lean body mass (low body fat levels) and a harmonious and aesthetically pleasing silhouette [4]. Peak interventions that alter the appearance of the figure usually take place in the final days before the competition. These include changes in exercise and dietary patterns, including manipulation of nutrient intake, particularly carbohydrates and water, and the use of dietary supplements such as diuretics [5–7]. The carbohydrate intervention consists of a severely restricted carbohydrate intake during the week before the competition, known as the fatigue period, followed by a short period of excessive consumption of a high carbohydrate diet to achieve supercompensation of glycogen [2]. The aim of this intervention is to maximise muscle glycogen content and thus increase muscle volume by combining the glycogen present with water in a ratio of 1 gram of glycogen to 3–4 grams of water. Another dietary change concerns the intake of water and sodium. Athletes practice what is known as water-loading, which consists of consuming between 4 and 12 litres of water a day, and then drastically reducing the water intake for 10 to 24 hours before the competition. The aim is to minimise the volume of subcutaneous water, giving the athlete a "dry" and sculpted appearance. Female athletes also seek to reduce the risk of abdominal bloating in order to achieve an optically smaller waist, which in turn helps to optimise body proportions, overall aesthetics and silhouette presentation at competitions [8–10].

The annual training and nutritional plan of a bodybuilder is divided into two phases. During the off-season, athletes focus on the regularity and intensity of their strength training and a positive caloric balance with a high protein intake (approximately 2.5–3.5 g/kg/day), which contributes to the growth of muscle mass. The aim of the strength training programme is to develop the correct body proportions which, combined with certain aesthetic features such as symmetry and pronounced muscular sculpture, will result in an optimal body appearance. The second, pre-competition phase, is a period during which athletes attempt to reduce fat mass (FM) to the lowest possible level while maintaining the low body mass (LBM) and silhouette appearance developed during the off-season. They achieve this through a combination of negative energy balance and rigorous aerobic training [8–11]. Unfortunately, low energy availability, the achievement of polyuria and forced water excretion from the body undoubtedly have a detrimental effect on an athlete’s body condition, which may lead to an increased risk of relative energy deficiency in sport (RED-S) symptoms [12]. Thus, participation in FP sports requires women to have a high lean body mass and extremely low fat mass, which in turn contributes to the risk of low energy availability (LEA) and the development of RED-S symptoms, a clinically diagnosed, multifactorial syndrome characterised by the accumulation of adverse health and performance outcomes resulting from exposure to problematic LEA [12].

Energy availability is the energy from food remaining and available for the optimal functioning of the body’s systems after taking into account energy expended during physical training. Unfortunately, there is often a situation where, intentionally or unintentionally, there is a mismatch between the amount of energy intake in relation to the the amount of the energy expenditure. LEA is an energy mismatch between energy intake and energy expenditure that leaves energy requirements unmet. There is an energy deficit and a lack of energy to maintain optimal body health. LEA can have mild and reversible effects (known as adaptable LEA), which is usually a short-term experience with minimal or no impact on the athlete’s health, or problematic LEA, during which there is a significant and prolonged deterioration of the athlete’s health and a lack of adaptability with the dysfunction of various body systems. Both eating disorders (mental illnesses clinically diagnosed), and disordered eating behaviours (restrictive eating, compulsive eating, excessive exercise, use of purgatives) lead to LEA [13–15].

The International Olympic Committee’s most recent consensus statement on RED-S, published in 2023, describes a wide range of physiological, psychological and performance-related impairments associated with LEA [12]. The consensus statement summarises several years of research on LEA and reports that inadequate energy intake relative to exercise leads to the impairment of bone health and the reproductive, cardiovascular, gastrointestinal and haematological function, energy/metabolism regulation, glucose and lipid metabolism, growth and development, neurocognitive function and skeletal muscle function as well as urinary incontinence, sleep disturbances and reduced immunity. It has also been found that mental health issues can either be a result of or precede RED-S. All of these health problems lead to reduced athletic performance or the athlete’s withdrawal from sport [12].

Female physique athletes are certainly an under-researched group of female athletes. This publication, to the best of the authors’ knowledge, is the first to assess LEA in female physique athletes using the LEAF-Q. The aim of this study was to assess the menstrual cycle disorders as well as injuries, and gastrointestinal problems among female physique athletes using LEAF-Q.

## Materials and methods

### Participants

The study involved 104 females who were physique athletes. The database used for writing this manuscript was collected courtesy of the publication’s co-author, a former silhouette sports athlete, who has contact with female athletes from this sporting environment. Verbal informed consent was obtained from all individual participant. Consent was documented as a response to an e-mail sent to each athlete. The research was conducted from the bagining of September 2020 to the end of February 2021. The study was carried out during the autumn-winter period, i.e. the preparation (non-competition) period, meaning that the athletes were in a period of caloric surplus and the training undertaken was mainly aimed at building up muscle mass, e.g. on the shoulders in order to improve body proportions and optically reduce the waistline. The mean age of the competitors was 26.77 ± standard deviation 5.76 years. The average height was 165.87±5.56cm, body weight was 58.43±6.30kg, however the athletes’ highest body weight at their current height was 65.30±10.88kg, and the lowest 50.08±5.39kg. The body mass index (BMI) was 21.25±1.86kg/m^2^. The average number of hours a week spent on training by the participants was 8.57±2.95. The types of training undertaken by the athletes were: strength training undertaken by 100% of the female athletes surveyed, cardio training by 66 athletes (63.46%), stationary cycling by 24 (23.08%), martial arts by 10 (9.62%), swimming by 6 (5.77%) and running by 4 (3.85%).

None of the women were using oral contraceptives during the study, however 13.46% of them stated that they had used oral contraceptives prior to the study. The reason for using oral contraception was to avoid pregnancy in 92.86% of the women and to regulate menstruation stops in 7.14%

Inclusion criteria for the study were regular training and participation in women’s physique competitions. The criteria for exclusion were the use of contraception during the study, a declaration of amenorrhea resulting from other known factors not related to physical activity, e.g., pregnancy, polycystic ovary syndrome, hysterectomy and the lack of a correctly completed questionnaire.

The study was performed in accordance with the ethical standards of the Declaration of Helsinki given ethics approval was obtained from the Bioethical Committee of the Andrzej Frycz Modrzewski Krakow University (Permission number KBKA/2/O/2020).

### Questionnaire

LEAF-Q was used in the study. It contains 25 questions arranged into three sections: injury, gastrointestinal function and reproductive function. Questions are scored according to a key provided with the questionnaire, in which a total score of ≥8 out of 25 questions indicates that the athlete is at risk of LEA [16].

### Statistical analyses

Statistical analyses were performed using the SPSS 27 software program (Version 27.0, IBM Corp., Armonk, NY, USA). Data were reported as means ± standard deviations and the chi-square test was used. The alpha level for all analyses was 0.05. As for effect sizes, for continuous variables, Cohen’s d was calculated as the effect size, and for categorical ones, the following was included: Cramer V. Following the guidelines for the interpretation of effect sizes, the following were assumed [17] for Cohen’s d: 0.2, 0.5, and 08; for Cramer V, with 1 df: 0.1, 0.3, and 0.5; and for Cramer V with 2 dfs: 0.07, 0.21, and 0.35 for small, medium, and large effect sizes, respectively.

### Power analysis

The sample of 104 subjects used in this research included all subjects who were available for research. A post-hoc power analysis was performed to determine what was the power for this sample. The analyses were performed by means of the G*Power software [18]. In the case of the chi-square test, the power for the small, medium, and large effect was 18%, 87%, and 99%, respectively. For the Mann-Whitney U test, the power for the three effect sizes was 13%, 56%, and 92%. Given this, it is clear that the sample size in the present research assured reasonable power for medium or large effects but not for small ones.

## Results

In most (89.42%) of the female physique athletes first menstruation had occurred naturally between 12 and 14 years of age, however in 10.58% it occurred over 15 years of age (Table 1). The majority of the athletes questioned, 78 women (75.00%), said that they had no problems with their monthly menstrual cycle, 25 women (24.04%) said that they did have problems with their monthly cycle, while one woman (0.96%) could not say whether she had problems with her monthly cycle or not and chose the following answer “I do not know”–this athlete was excluded from further analyses. The majority of women (94.87%) reported having had their last menstrual period 0–4 weeks ago and in line with this, the same percentage of women admitted that their monthly cycle was regular, it occurred every 28th to 34th day. In addition, the majority of women surveyed (96.15%) reported that bleeding lasted between 3 to 9 days or more. When asked how many monthly cycles they had had in the last year, 62.82% said 12 or more, 33.33% said 9–11 and 3.85% said only 6–8. Interestingly, of the 25 athletes who said they had irregular menstrual cycles, 57.69% had had their last menstrual period 2–3 months before the date of the survey, 30.77% had had their last menstrual period 6 or more months ago, and 11.54% had had their last menstrual period 4–5 months ago. It should also be noted that only 41.35% of the sportswomen had never experienced a time when their monthly cycle had stopped for more than three months or longer (besides during pregnancy). Of the 58.65% who had experienced this situation, it had happened to 43.27% before this study and 15.38% were experiencing it at the time of this study.

**Table 1:** The most important questions of LEAF-Q and the numerical answers provided by the female physique athletes, and responses to key components of LEAF-Q in relation to menstruation (chi-square tests), and analysis of female athletes tested according to the number of points scored on the LEAF-Q questionnaire (score ≤ 8 points, or score ≥ 8 points).

**Low Energy Availability in Females Questionnaire (questions)**	**Frequency (N)**	**Percent (%)**
The most important questions of LEAF-Q and the numerical answers provided by the female physique athletes		
**Injuries**		
Absences from your training, or participation in competitions during the last year due to injuries		
No, not at all	75	72.12
Yes, once or twice	19	18.27
Yes, three or four times	7	6.73
Yes, five times or more	3	2.88
Total	104	100.00
Numbers of days absence from training or participation in competition due to injuries		
1–7 days	12	41.38
8–14 days	8	27.59
15–21 days	5	17.24
22 days or more	4	13.79
Total	29	100.00
**2. Gastro intestinal function**		
Do you feel gaseous or bloated in the abdomen, also when you do not have your period?		
Yes, several times a day	21	20.20
Yes, several times a week	22	21.15
Yes, once or twice a week or more seldom	26	25.00
Rarely or never	35	33.65
Total	104	100.00
Do you get cramps or stomach ache which cannot be related to your menstruation?		
Yes, several times a day	8	7.69
Yes, several times a week	5	4.81
Yes, once or twice a week or more seldom	26	25.00
Rarely or never	65	62.50
Total	104	100.00
How often do you have bowel movements on average?		
Several times a day	14	13.46
Once a day	71	68.27
Every second day	17	16.35
Twice a week	2	1.92
Total	104	100.00
**3. Menstrual function**		
How old were when you had your first period?		
from 11 (or younger) to 14 years	93	89.42
15 years or older	11	10.58
Total	104	100.00
Do you have normal menstruation?		
YES	78	75.00
NO	25	24.04
I do not know	1	0.96
Total	104	100.00
When did you have your last period?		
0–4 weeks ago	74	94.87
1–2 months ago	4	5.13
Total	78	100.00
If yes, are your periods regular? (Every 28th to 34th day)		
Yes, most of the time	74	94.87
No, mostly not	4	5.13
Total	78	100.00
If yes, for how many days do you normally bleed?		
1–2 days	3	3.85
between 3 to 9 days or more	75	96.15
Total	78	100.00
Have you ever had problems with heavy menstrual bleeding?		
NO	104	100
If yes, how many periods have you had during the last year?		
12 or more	49	62.82
9–11	26	33.33
6–8	3	3.85
Total	78	100.00
If not, when did you have your last period?		
2–3 months ago	14	57.69
4–5 months ago	3	11.54
6 months ago or more	8	30.77
Total	25	100.00
Have your periods ever stopped for 3 consecutive months or longer (besides pregnancy)?		
No, never	43	41.35
Yes, it has happened before	45	43.27
Yes, that’s the situation now	16	15.38
Total	104	100.00
Do you experience that your menstruation changes when you increase your exercise intensity, frequency or duration?		
YES	64	61.54
NO	40	38.46
Total	104	100.00
If yes, how?	
I bleed less	36	56.25
I bleed more	10	15.63
My menstruations stops	18	28.12
Total	64	100.00

Responses to key components of LEAF-Q in relation to menstruation (chi-square tests)				
	**MENSTRUATION**			
	**No problem**		**Problem**	
**1. Injuries**				
Absences from your training, or participation in competitions during the last year due to injuries				
***chi*** ^***2***^ **(3) = 2.95; *p* = .400, Cramer’s *V* = .17**				
	**N**	**%**	**N**	**%**
No, not at all	58	74.36	16	64.00
Yes, once or twice	12	15.38	7	28.00
Yes, three or four times	5	6.41	2	8.00
Yes, five times or more	3	3.85	0	0.00
Total	78	100.00	25	100.00
Numbers of days absence from training or participation in competition due to injuries				
***chi*** ^***2***^ **(3) = 2.17; *p* = .537, Cramer’s *V* = .27**				
1–7 days	7	35.00	5	55.56
8–14 days	7	35.00	1	11.11
15–21 days	3	15.00	2	22.22
22 days or more	3	15.00	1	11.11
Total	20	100.00	9	100.00
**2. Gastro intestinal function**				
Do you feel gaseous or bloated in the abdomen, also when you do not have your period?				
***chi*** ^***2***^ **(3) = 3.13; *p* = .372, Cramer’s *V* = .17**				
Yes, several times a day	17	21.79	3	12.00
Yes, several times a week	14	17.95	8	32.00
Yes, once or twice a week or more seldom	19	24.36	7	28.00
Rarely or never	28	35.90	7	28.00
Total	78	100.00	25	100.00
Do you get cramps or stomach ache which cannot be related to your menstruation?				
***chi*** ^***2***^ **(3) = 13.57; *p* = .004, Cramer’s *V* = .36**				
Yes, several times a day	7	8.97	0	0.00
Yes, several times a week	4	5.13	1	4.00
Yes, once or twice a week or more seldom	13	16.67	13	52.00
Rarely or never	54	69.23	11	44.00
Total	78	100.00	25	100.00
How often do you have bowel movements on average?				
***chi*** ^***2***^ **(3) = .94; *p* = .815, Cramer’s *V* = .10**				
Several times a day	10	12.82	4	16.00
Once a day	54	69.23	16	64.00
Every second day	13	16.67	4	16.00
Twice a week	1	1.28	1	4.00
Total	78	100.00	25	100.00
**3. Menstrual function**				
How old were when you had your first period?				
***chi*** ^***2***^ **(1) = .25; *p* = .618, Cramer’s *V* = .05**				
from 11 (or younger) to 14 years	69	88.46	23	92.00
15 years or older	9	11.54	2	8.00
Total	78	100.00	25	100.00
Have your periods ever stopped for 3 consecutive months or longer (besides pregnancy)?				
***chi*** ^***2***^ **(2) = 61.90; *p* < .001, Cramer’s *V* = .76**				
No, never	41	52.56	1	4.00
Yes, it has happened before	37	7.44	8	32.00
Yes, that’s the situation now	0	.00	16	64.00
Total	78	100.00	25	100.00
Do you experience that your menstruation changes when you increase your exercise intensity, frequency or duration?				
***chi*** ^***2***^ **(1) = 5.90; *p* = .015, Cramer’s *V* = .24**				
Yes	44	56.41	20	80.00
No	34	43.59	5	20.00
Total	78	100.00	25	100.00
If yes, how?				
***chi*** ^***2***^ **(2) = 6.90; *p* = .032, Cramer’s *V* = .33**				
I bleed less	28	63.64	8	40.00
I bleed more	8	18.18	2	10.00
My menstruations stops	8	18.18	10	50.00
Total	44	100.00	20	100.00
Analysis of female athletes tested according to the number of points scored on the LEAF-Q questionnaire (score ≤ 8 points, or score ≥ 8 points)				
	**TOTAL POINTS**			
	**score ≤ 8 points (n = 56)**		**score ≥ 8 points (n = 48)**	
**1. Injuries**				
Absences from your training, or participation in competitions during the last year due to injuries				
***chi*** ^***2***^ **(3) = 9.47; *p* = .024, Cramer’s *V* = .30**				
	**N**	**%**	**N**	**%**
No, not at all	47	83.93	28	58.33
Yes, once or twice	7	12.50	12	25.00
Yes, three or four times	1	1.79	6	12.50
Yes, five times or more	1	1.79	2	4.17
Total	56	100.00	48	100.00
Number of days absence from training or participation in competition due to injuries				
***chi*** ^***2***^ **(3) = 7.78; *p* = .051, Cramer’s *V* = .52**				
1–7 days	4	44.44	8	40.00
8–14 days	5	55.56	3	15.00
15–21 days	0	.00	5	25.00
22 days or more	0	.00	4	20.00
Total	9	100.00	20	100.00
**2. Gastro intestinal function**				
Do you feel gaseous or bloated in the abdomen, also when you do not have your period?				
***chi*** ^***2***^ **(3) = 20.28; *p* < .001, Cramer’s *V* = .44**				
Yes, several times a day	5	8.93	16	33.33
Yes, several times a week	7	12.50	15	31.25
Yes, once or twice a week or more seldom	18	32.14	8	16.67
Rarely or never	26	46.43	9	18.75
Total	56	100.00	48	100.00
Do you get cramps or stomach ache which cannot be related to your menstruation?				
***chi*** ^***2***^ **(3) = 20.87; *p* < .001, Cramer’s *V* = .45**				
Yes, several times a day	1	1.79	7	14.58
Yes, several times a week	1	1.79	4	8.33
Yes, once or twice a week or more seldom	8	14.29	18	37.50
Rarely or never	46	82.14	19	39.58
Total	56	100.00	48	100.00
How often do you have bowel movements on average?				
***chi*** ^***2***^ **(3) = 9.69; *p* = .021, Cramer’s *V* = .31**				
Several times a day	6	10.71	8	16.67
Once a day	45	80.36	26	54.17
Every second day	5	8.93	12	25.00
Twice a week	0	.00	2	4.17
Total	56	100.00	48	100.00
How would you describe your normal stool?				
***chi*** ^***2***^ **(2) = 7.58; *p* = .023, Cramer’s *V* = .27**				
Normal (soft)	54	96.43	38	79.17
Diarrhoea-like (watery)	1	1.79	4	8.33
Hard and dry	1	1.79	6	12.50
Total	56	100.00	48	100.00
**3. Menstrual function**				
How old were when you had your first period?				
***chi*** ^***2***^ **(1) = .35; *p* = .555, Cramer’s *V* = .06**				
from 11 (or younger) to 14 years	51	91.07	42	87.50
15 years or older	5	8.93	6	12.50
Total	56	100.00	48	100.00
When did you have your last period?				
***chi*** ^***2***^ **(1) = 4.20; *p* = .040, Cramer’s *V* = .23**				
0–4 weeks ago	54	98.18	20	86.96
1–2 months ago	1	1.82	3	13.04
Total	55	100.00	23	100.00
Are your periods regular? (Every 28th to 34th day)				
***chi*** ^***2***^ **(1) = 10.08; *p* = .001, Cramer’s *V* = .36**				
Yes, most of the time	55	100.00	19	82.61
No, mostly not	0	.00	4	17.39
Total	55	100.00	23	100.00
For how many days do you normally bleed?				
***chi*** ^***2***^ **(1) = .02; *p* = .882, Cramer’s *V* = .02**				
1–2 days	2	3.64	1	4.35
3–9 days or more	53	96.36	22	95.65
Total	55	100.00	23	100.00
How many periods have you had during the last year?				
***chi*** ^***2***^ **(2) = 8.38; *p* = .015, Cramer’s *V* = .33**				
12 or more	40	72.73	9	39.13
9–11	14	25.45	12	52.17
6–8	1	1.82	2	8.70
Total	55	100.00	23	100.00
When did you have your last period?				
***chi*** ^***2***^ **(2) = .76; *p* = .683, Cramer’s *V* = .17**				
2–3 months ago	1	100.00	14	56.00
4–5 months ago	0	.00	3	12.00
6 months ago or more	0	.00	8	32.00
Total	1	100.00	25	100.00
Have your periods ever stopped for 3 consecutive months or longer (besides pregnancy)?				
***chi*** ^***2***^ **(2) = 26.53; *p* < .001, Cramer’s *V* = .51**				
No, never	34	60.71	9	18.75
Yes, it has happened before	21	37.50	24	50.00
Yes, that’s the situation now	1	1.79	15	31.25
Total	56	100.00	48	100.00
Do you experience menstruation changes when you increase your exercise intensity, frequency or duration?				
***chi*** ^***2***^ **(1) = 9.82; *p* = .002, Cramer’s *V* = .31**				
Yes	25	44.64	36	75.00
No	31	55.36	12	25.00
Total	56	100.00	48	100.00
If yes, how?				
***chi*** ^***2***^ **(2) = 6.71; *p* = .035, Cramer’s *V* = .32**				
I bleed less	19	70.37	17	45.95
I bleed more	5	18.52	5	13.51
My menstruations stops	3	11.11	15	40.54
Total	27	100.00	37	100.00

The study showed that female physique athletes with menstrual disorders were younger (problem age 23.80±3.70 years vs. no-problem 27.72±6.02 years, p<0.001) and undertook more training per week (problem 9.96±2.81 vs. no-problem 8.14±2.88 hours of training per week, p<0.002) than those with no menstrual disorders (Table 2). Furthermore, women who had a menstrual disorder were significantly more likely (p<0.01) to suffer from cramps or stomach ache unrelated to menstruation compared to women without such problems. They were also more likely to have no menstruation for 3 consecutive months or longer (besides during pregnancy) (p<0.001) and there were more changes in menstruation when the exercise intensity, frequency or duration was increased (p = 0.015) (Table 1). These changes were most often characterised by the absence of a monthly cycle (p = 0.032). It should be noted, however, that female athletes with a normal monthly cycle also reported changes after an increase in the intensity, frequency or length of training—mainly bleeding that lasted for less time (fewer days) than usual. This problem was reported by 63.64% of women with a normal monthly cycle vs. 40.00% of women with a menstrual disorder, but there were also women who reported bleeding lasting more days than usual—18.18% of athletes with a normal monthly cycle vs. 10% of those with an abnormal monthly cycle (Table 1).

**Table 2:** Differences between female physique athletes who have disorders of the monthly cycle (problem (P), n = 25) and without such problems (no problem (NP), n = 78).

	Mean		Median		Standard deviation				
	No problem (NP) *n* = 78	Problem (P) *n* = 25	NP	P	NP	P	U	p	Cohen’s d
Age [years]	27.72	23.80	27.00	23.00	6.02	3.70	534.50	**.001**	.71
Height [cm]	165.88	165.72	166.00	165.00	5.68	5.40	942.00	.799	.03
Weight [kg]	58.34	58.80	58.00	58.00	6.01	7.34	972.00	.982	-.07
BMI [kg/cm^2^]	21.23	21.38	20.86	20.90	1.81	2.06	899.00	.559	-.08
The highest weight with your present height [kg]	65.35	65.36	63.50	63.00	11.21	10.19	969.00	.963	.00
The lowest weight with your present height [kg]	50.32	49.30	50.00	49.00	5.61	4.82	886.00	.493	.19
Number of weekly training sessions [hours]?	8.14	9.96	8.00	10.00	2.88	2.81	572.00	**.002**	-.64

The women athletes’ responses to the questionnaire were scored using a scoring key, where a score of 8 points represented the dividing or borderline. When a competitor scored ≥ 8 points this indicated that she was at risk of low energy availability and, as a consequence, may be at risk of developing the Female Athlete Triad and RED-S groups of symptoms. It was found that the group of female athletes who scored less than 8 points were older (p<0.018) and trained less frequently (p<0.028) than the group of athletes who scored 8 or more (Table 3). Furthermore, the statistical significance was p<0.024 indicating that women in the group that scored over 8 points according to the LEAF-Q questionnaire, were more frequently absent from training due to injury and their absences lasted longer than the women in the group with lower scores according to the scoring key p<0.05. Moreover, those women, in the 8 points and above group, had significantly statistically more frequent gastrointestinal problems, including feeling gaseous or bloated in the abdomen more frequently, or when they did not have period, getting cramps or stomach ache which were not related to menstruation (p<0.001). They also had less frequent bowel movements (p<0.021), and their stools were significantly more often described as hard and dry (p<0.023) (Table 1). With regard to the monthly cycle, those in the 8 points and above group were significantly more likely to have had their period within one to two months of the study (p<0.040) and were less likely to declare that their menstruation was regular, i.e. every 28–34 days (p<0.001). In answer to the question: how many menstrual periods had they had in the last year, women in the less than 8 points group more often stated 12 or more, while responses of 9–11 times and 6–8 times occurred more frequently in the 8 points and more group (p<0.015) (Table 1). In the group of athletes who scored 8 and above according to the scoring key, periods stopped significantly more often for 3 consecutive months or longer (p<0.001), and menstruation changes occurred, including a significantly shorter bleeding time (lasting fewer days than usual) or the complete disappearance of the monthly cycle (p<0.035) when there was an increase in intensity, frequency or duration of exercise (p<0.002).

**Table 3:** Analysis of the studied women according to total scores on the LEAF-Q questionnaire (cut-off score ≥ 8 points).

	Mean		Median		Standard deviation				
	score ≤ 8 points	score ≥ 8 points	score ≤ 8 points	score ≥ 8 points	score ≤ 8 points	score ≥ 8 points	U	p	Cohen’s d
Age [years]	28.11	25.21	27.00	24.50	6.48	4.35	982.50	**.018**	.52
Height [cm]	165.02	166.85	165.00	166.00	5.56	5.46	1102.50	.114	-.33
Weight [kg]	57.54	59.48	58.00	58.50	5.92	6.62	1147.00	.198	-.33
BMI [kg/cm^2^]	21.18	21.34	21.01	20.81	1.90	1.83	1316.00	.855	-.34
The highest weight with your present height [kg]	65.39	65.19	63.00	64.00	12.76	8.30	1211.50	.386	-.31
The lowest weight with your present height [kg]	49.76	50.47	49.50	50.00	5.62	5.15	1172.00	.261	-.09
Number of weekly training sessions [hours]?	8.04	9.19	8.00	10.00	2.56	3.26	1010.00	**.028**	.02

## Discussion

Female athletes are particularly susceptible to LEA in physique sports, where physical appearance (silhouette) is of paramount importance and subject to assessment by the judges [19]. Negative caloric balance and prolonged periods of energy deprivation lead to serious health consequences in athletes, resulting in impaired physiological and/or psychological functioning of the entire body [12].

This study is the first to examine a group of female physique athletes with the use of LEAF-Q. It showed that approximately 24% of these athletes did not have a regular monthly cycle. However, it should be noted that disturbances to the monthly cycle lasting more than three months and which occurred prior to this study were reported by about 43% of the women, while approximately 15% of the athletes reported that this was happening during this study. These figures suggest that LEA actually affects more women than is reported when they are asked about both the normality and regularity of their monthly cycle. Looking at the results in relation to the cut-off score given by the LEAF questionnaire, it was found that approximately 46% of the women scored ≥ 8 points. This indicates that almost half of the female athletes in the study group were at direct risk of LEAs and, consequently, the dysfunctions associated with RED-S. This research also showed that, in the group of women with both normal and disturbed menstrual cycles, an increase in the intensity, frequency or duration of exercise corresponded to significant changes in monthly bleeding, mainly that it was shorter. In their study Meng et al. [20] came to similar conclusions. These authors noted that, in elite athletes, increased training frequency and intensity leads to increased energy expenditure, which in turn increases the risk of LEA. In the study by Folscher et al. [21] it was reported that up to 50% of women who maintain a normal regular monthly cycle experience changes in monthly bleeding when the intensity of the physical activity undertaken increases. These findings are consistent with the study by Micklesfield et al. [22] and Duckham et al. [23], which indicated that monthly cycle disorders occurred in 43–78% of athletes when their training intensity increased.

Dysfunctions in the regularity of the menstrual cycle are common in women involved in physique athletes sports. When involved in a sport that focuses on figure modelling, a number of factors come into play that can contribute to these disorders. These include the high frequency and intensity of training, strict diets, low body fat levels and high levels of stress associated with competition [24–26]. It should be noted that our own research showed no significance between the body mass index and weight of the athletes and the total number of points regarded as borderline according to the LEAF questionnaire scores. These results can probably be explained by the fact that the study was carried out in the autumn/winter period, when most athletes were outside the competition season, when there is a tendency to increase caloric balance and to gain weight. Disruption of the menstrual cycle is also a cause of oestrogen deficiency, which leads to a decrease in bone mineral density, and thus an increased risk of stress fractures [24–26]. The results of our own study showed that 36% of the athletes, i.e. nine out of twenty-five women who had a menstrual cycle disruption, had suffered a sports injury in the previous year. Other studies have also shown that, when it comes to aesthetic sports, the incidence of LEA is more common in elite athletes (55.8%) than in recreational athletes (35.1%) [19]. In a study conducted by Logue et al. [27] however, Irish athletes, risk of LEA was 1.7 and 1.8 times more likely in international and provincial/inter-county athletes compared to those who were recreationally active. Interesting results were also reported in a study of elite Australian athletes [28], in which 53% of the athletes surveyed reported LEA-related symptoms, on the basis of LEAF questionnaire scores. In a study of 425 female aesthetic athletes by Beals et al. [29] 31% of the athletes reported irregular monthly cycles.

Our own research showed that the group of women who scored less than 8 on the questionnaire scores were older and trained less frequently than the group of athletes who scored 8 and above. This suggests that younger women involved in physique sports may be particularly susceptible to monthly cycle disorders. Similar findings were reported by Loucks [30] who found that women aged 25–40 years who followed a controlled calorie-restricted exercise programme had a lower risk of monthly cycle disorders than women aged 14–18 years. As noted by De Souza et al. [25] the current trend of increasing numbers of women taking up competitive sport at a young age is associated with a worrying increase in the female athlete triad.

Due to the nature of physique sports, female athletes manipulate their intake of specific foods exposing themselves to LEA. Reduction, the process by which very low levels of body fat are attained, is achieved through negative caloric balance and intense aerobic training [13–15]. Our own research shows that the body weight of the athletes could vary from a high of 65.36 ± 10.19 kg to a low of 49.30 ± 4.82 kg. Such a difference is undoubtedly significant for the athlete’s body, especially as such significant changes in body weight occurred when the height of the athletes studied remained unchanged. Studies by Hinton et al. [31] and Keay et al. [32] confirm that deliberate carbohydrate restriction and negative caloric balance are associated with a risk of LEA. A study by Burke et al. [33], also suggests that the long training hours typical of athletes competing at the highest level may result in restricted access to food intake, further increasing the risk of energy deficiency. In aesthetic sports, athletes are required to adhere strictly to a set training schedule and dietary recommendations. The results of the study by Van Durme et al. [34] clearly show that female figure skaters and ballet dancers, i.e. women who engage in physical activities that require a slender figure, are more prone to nutritional problems, have higher levels of bulimia symptoms, and experience greater weight and appearance concerns than their peers who do not engage in these types of activities.

Our own research has shown that women who participate in physique sports often experience gastrointestinal problems. Approximately 66% of women said they experienced uncomfortable gas or bloating that was not related to their period. Additionally, around 37% of athletes reported experiencing abdominal cramps and pains again not related to their menstruation. These results may indicate that a calorie-restricted diet, psychological stress and excessive physical activity, factors that are inherent characteristics of aesthetic sports, have a negative impact on the digestive system. It is likely that the manipulation of carbohydrates, water and sodium, i.e. the dietary practices used for a week or two prior to competition by female figure sports athletes, may also have a significant impact on the occurrence of gastrointestinal problems [35]. It is worth noting that similar findings were reported in a study by Condo et al. [36], who found that 51.8% of female players in the Australian Football League experienced gas or bloating problems, while 32.2% experienced cramping or abdominal pain unrelated to menstruation. In a study by Fahrenholtz et al. [37] athletes who reported food intolerances had higher overall scores on the LEAF-Q than those who did not report such problems. It should be noted, however, that the information on food intolerance was provided by the athletes themselves, which may raise some questions about the causes of gastrointestinal complaints. It is not clear whether these problems are directly due to food intolerance or to changes in the gastrointestinal tract caused by energy deprivation. In our study, a significant association was observed between scoring ≥8 on the LEAF questionnaire and the occurrence of non-menstrual related symptoms such as gas, bloating, abdominal pain and cramping.

Knowledge of the principles of sports nutrition and the correct choice of training are extremely important in order to maintain an athlete’s optimal health, prevent energy deficiencies and prepare the athlete’s body for the high level of physical exertion. However, in physique (aesthetic) sports, dietary restrictions, which usually result in a negative caloric balance, combined with intensive training, cause health problems that are manifested by a decline in the athlete’s performance [12]. Biological processes related to health, growth, reproduction and activity compete for the body’s limited energy resources [38]. The occurrence of energy depletion, or energy commitment to a process, results in the transfer of energy from processes that are temporarily redundant or reducible, such as reproductive functions. However, in some sports-related practices, athletes intentionally or unintentionally expose the body to problematic LEAs. This results in a situation where the body is unable to adapt to energy deprivation, which in turn is associated with negative effects on various body systems and ultimately leads to an overall deterioration in the health of the athlete [12].

The prevention of LEA in athletes is possible by ensuring that the amount of calories they consume is in line with the body’s current needs and, if LEA occurs, by treating the athlete on the basis of increased energy availability. However, in the case of physique sports, especially in the pre-competition period, this is unrealistic and even contrary to what female physique athletes want to achieve. During this period, female athletes are focusing on losing weight and keeping their body fat as low as possible. This process allows them to achieve a lean and proportionate physique that will ultimately be assessed by a panel of judges during the competition.

### Limitations

The main limitation of this study was that it was not possible to perform a body composition analysis and accurately determine the difference in body fat between the competition and non-competition periods. The study was carried out at only one time point, the preparation period; it would have been interesting to monitor the results of the LEAF-Q questionnaire in the preparation and competition phases. It would also have been beneficial to monitor the levels of selected hormones and to determine the individual energy balance of the athletes.

## Conclusion

About 46% of the women studied had a borderline LEAF-Q score (≥ 8 points), meaning that almost half of the women in the study group were at immediate risk of LEA. Using the LEAF questionnaire, menstrual cycle disorders were detected in 24% of the athletes; however, menstrual cycle disorders lasting more than three months and occurring prior to the study were reported by about 43% of the women, and about 15% of the athletes reported that this had occurred during the study. Women who had menstrual cycle disorders were younger and did more training per week compared to the women who did not report a menstrual cycle disorder. Missed training due to injury was shown to be more common in women in the score ≥ 8 group.

Women who participate in physique sports often have gastrointestinal problems. About 66% of the women experienced unpleasant symptoms of excessive gas or bloating that were not related to their period. In addition, around 37% of athletes reported experiencing abdominal cramps and pains that were not related to their menstruation. These changes were significantly more common in the group with a score ≥ 8.

## Supporting information

S1 Dataset (XLSX)
